# The function of supplemental foods for improved crop establishment of generalist predators *Orius insidiosus* and *Dicyphus hesperus*

**DOI:** 10.1038/s41598-018-36100-0

**Published:** 2018-12-12

**Authors:** Roselyne M. Labbé, Dana Gagnier, Ana Kostic, Les Shipp

**Affiliations:** Harrow Research and Development Centre, Agriculture and Agri-Food Canada, Harrow, Ontario Canada

## Abstract

As with many biological control agents, generalist predators rarely survive prolonged periods of prey scarcity. Towards improving crop establishment of two major predators used in North America, *Orius insidiosus* and *Dicyphus hesperus*, this study examined the role of supplemental foods in achieving greater predator survival and faster development. In controlled environment trials, developmental time and survival were compared for predators offered diets including *Ephestia* eggs, *Artemia* cysts, *Typha* pollen, or combinations of these. Nymphal developmental time was significantly shorter and survival greater for both predators reared on diets that included *Ephestia* eggs. Interestingly, *D*. *hesperus* could successfully complete nymphal development on *Artemia* cysts whereas *O. insidiosus* could not, alluding to fundamental physiological differences between these predators. In greenhouse assays, *D*. *hesperus* was more abundant after six weeks when offered diets that included *Ephestia* eggs either alone or in combination with pollen or *Artemia* cysts relative to other diets. In contrast, only diets of *Ephestia* eggs, *Typha* pollen or their combination could significantly increase *O*. *insidiosus* crop abundance relative to the unfed control. Together, this work highlights important differences in the relative values of supplemental foods for generalist predators used in crop protection. It is also meaningful in guiding biocontrol practitioners globally in the rapidly growing sector of greenhouse vegetable production.

## Introduction

One of the modern challenges of global food production systems consists of meeting the demands for increased productivity while maintaining their environmental and economic sustainability^1,2^. In lockstep, the quantity of food produced in protected environments has rapidly increased in many parts of the world as it offers producers an added degree of protection against abiotic and biotic stresses. Furthermore, biological control, in which predators, parasitoids and pathogens are applied to crops for the suppression of pests, is well recognized as an effective means for achieving crop protection in these environments^3^. Yet improvements are still needed to ensure the economic sustainability and adaptability of existing biological control programs.

Until recently, greenhouse biological control has consisted predominantly of the inundative release of specialist natural enemies to attack crop pests. While effective, specialist agents tend to rapidly disappear from crops once pest prey resources are mostly expended, requiring frequent and costly reintroductions. In contrast, generalist agents play important, if not yet fully understood^4^ or appreciated^5^, roles in mediating the threat of pests in these highly simplified monoculture agroecosystems^6^. Furthermore, increasing the number of linkages in a greenhouse trophic environment has the potential to better mediate crop pest populations. The addition of generalist and omnivorous predators within such a system has the added benefit of achieving a broader spectrum and longer lasting pest suppression as these agents are able to exploit not only multiple pest types but also other arthropod prey and plant food resources with which to breach periods of prey scarcity^7^. As such, generalists can be established preventatively on a crop where they are able to rapidly dampen the incidence of potential pest outbreaks^8,9^. In particular, multiple generalist predatory agents including mirids^10,11^, anthocorids, and mites^12,13^, are now considered integral parts of greenhouse biological control programs. Omnivorous predatory mirids such as *Macrolophus* spp. (Heteroptera: Miridae) and *Dicyphus* spp. (Heteroptera: Miridae) for instance, who derive nutrients and water from host plants^14^, are excellent predators of many common greenhouse pests such as thrips, whiteflies, mites, aphids and other soft bodied arthropods^11,15–17^. Indeed, in the western Mediterranean basin, natural colonization of greenhouse crops by mirids *Dicyphus tamaninii* (Wagner) and *Macrolophus caliginosus* (Wagner) is regularly observed^18^. In a similar way, the anthocorid predators within the genus *Orius* (Hemiptera: Anthocoridae), including *O*. *majusculus* (Reuter) also naturally colonize and reproduce on greenhouse crops in northern Europe where they attack pests such as thrips^19^. These examples highlight the intrinsic suitability of generalist predatory bugs for biological control of multiple crop pests^18^. In North America the generalist predators *Dicyphus hesperus* (Knight) and *Orius insidiosus* (Say) take on a similar function in combatting both established as well as potentially invasive pest species^20^, and it is therefore important to determine how best to optimize their crop presence despite periods of prey scarcity^21^.

For anthocorid and mirid predators, food supplementation with plant or alternate prey resources can greatly improve their long-term crop establishment and contribute to a more sustainable pest management strategy^10,22–25^. Traditionally, eggs of the Mediterranean flour moth, *Ephestia kuehniella* have been widely used as a food source to maintain populations of predatory bugs^26–29^. However, due to the technical challenge of producing this food source^20^, its cost is generally considered prohibitive for wide scale application in crop food supplementation. As such, the search for less costly yet nutritionally suitable alternatives has led to the investigation of pollen and *Artemia* cysts as food sources with potential value.

Pollen is also an important food source for a number of flower dwelling anthocorid and some mirid species and its availability on a crop can improve the persistence of these natural enemies^19^. For instance, the fecundity of *Orius albidipennis* (Reuter) is significantly greater when this predator is offered a combination of pollen and eggs of *Ephestia kuehniella* relative to *Ephestia* eggs alone^23^. *Orius insidiosus* and *Macrolophus pygmaeus* (Rambur) are also both known to develop to adulthood when reared on pollen alone^10,30^, and *O. insidiosus* population growth can improve considerably when offered bee collected or *Ricinus spp*. (Malpighiales: Euphorbiaceae) pollen^29^. However, response to pollen is variable even among members of well recognized pollen feeding predatory species and should be formally evaluated^19^.

Likewise, the cysts of brine shrimp, *Artemia spp*. (Anostraca: Artemiidae), have also been shown to provide nutritional benefit to some, but not all generalist predators. *Orius laevigatus* (Fieber) females fed on the cysts of *A. fransiscana* (Leach), had a similar fecundity relative to such predators offered *Ephestia kuehniella* Zeller (Lepidoptera: Pyralidae) eggs^31^. In biochemical analyses, *Artemia* cysts were shown to contain equal or greater amounts of protein relative to eggs of *Ephestia*, though fatty acid content was nearly three times lower in *Artemia* cysts relative to *Ephestia* eggs^24^. These attributes may incur different consequences for predators on a species basis and may affect factors such as the rate of nymphal development, survival, as well as predator fecundity. In laboratory assays, nymphal survival of *O. laevigatus* was no different between individuals reared on decapsulated *Artemia* cysts relative to those reared on *E*. *kuehniella* eggs^24^. However, developmental time of *O. laevigatus* was up to 1.6 days longer than individuals reared on *E*. *kuehniella*^24^. In this study, laboratory and small cage greenhouse assays were conducted to better understand the relative suitability of *Artemia* cysts, cattail pollen *Typha latifolia L*. (Poales: Typhaceae), eggs of *Ephestia kuehniella* or combinations of these to the nymphal development time, survival and crop establishment of two generalist predators, *O. insidiosus* and *D. hesperus* whom are frequently used for biological control of pests on greenhouse crops in North America. The implications for these results are particularly relevant to the development of reliable augmentative biological control strategies involving these species in North America and their economic feasibility is further discussed in light of these results.

## Results

### Laboratory developmental assays

#### *Dicyphus hesperus*

**Figure 1:**
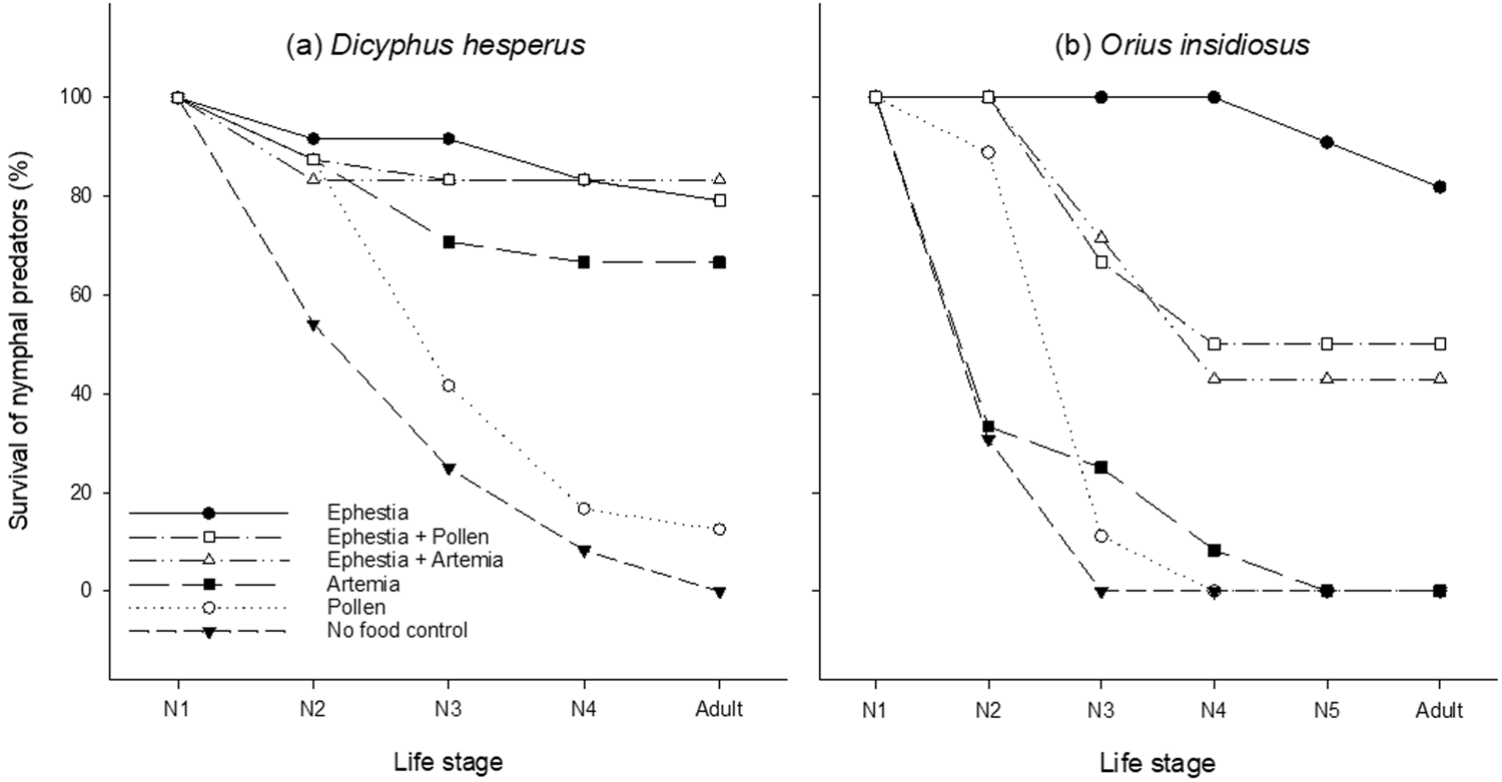
Percent nymphal predator survival to adulthood. Percent survival of *Dicyphus hesperus* (N = 24) (**a**) and *Orius insidiosus* (N = 12) (**b**) nymphal predators at each developmental life stage when reared on different supplemental food treatments or no-food control.

In laboratory trials, the percent survival of developing *D. hesperus* nymphs varied considerably among predators offered one of the six diet treatments tested, with 83.3% of individuals completing nymphal development on a diet of *Ephestia* eggs + *Artemia* cysts, 79.2% on a diet of *Ephestia* eggs alone and *Ephestia* + *Typha* pollen. On an *Artemia* cyst diet, 66.7% nymphs survived to adulthood compared to only 12.5% on pollen and no survival under control unfed conditions (Fig. 1a). Nymphal developmental time of *D*. *hesperus* predators also differed significantly based on supplemental food type (F_4,29_ = 4.95, P = 0.0032). Individuals reared on *Ephestia* eggs alone or combined with either *Typha* pollen or *Artemia* cysts developed in the shortest amount of time, followed by those reared on *Artemia* alone, and last on pollen alone (Table 1).

**Table 1:** Time required for *Dicyphus hesperus* predators to complete each developmental instar when reared on varying food sources.

Treatment	Developmental time (mean days ± SEM)				
	N1	N2	N3	N4	Total N1- adult
Pollen + *Ephestia*	3.3 ± 0.53	4.2 ± 0.56	4.8 ± 0.52	5.3 ± 0.50	17.3 ± 0.73 a
*Artemia* + *Ephestia*	3.4 ± 0.44	4.3 ± 0.26	4.6 ± 0.43	5.5 ± 0.47	17.7 ± 0.58 ab
*Ephestia*	3.5 ± 0.43	5.1 ± 0.36	4.1 ± 0.40	5.9 ± 0.46	18.3 ± 0.83 ab
*Artemia*	4.8 ± 0.25	4.7 ± 0.59	5.3 ± 0.50	5.9 ± 0.61	20.7 ± 0.78 bc
*Typha* pollen	2.0 ± 2.0	4.5 ± 1.5	16.5 ± 13.5	6.0 ± 4	29.0 ± 9.0 c
No food control	3.3 ± 0.72	5.8 ± 1.5	4.5 ± 0.5	NA	NA

Means for total developmental time as shown in the last column were compared using Tukey’s HSD; α = 0.05; (N = 24).

#### *Orius insidiosus*

In a first round of laboratory assays, we evaluated the suitability of various food sources to support the survival and development of *O*. *insidiosus* from newly emerged nymphs to adulthood. In general, the survival of *O*. *insidiosus* varied widely for each of the different food sources tested. For instance, survival to adulthood was greatest (82.0%) for *O*. *insidiosus* reared on eggs of *E. kuehniella* followed by those reared on a diet containing both *Ephestia* eggs and pollen (50.0%) or *Ephestia* eggs and *Artemia* cysts (42.9%) at a ratio of 1:5 (Fig. 1b). In contrast, nymphs of the unfed control, pollen or *Artemia* *spp*. cyst treatments failed to survive past the second, third or fourth instars respectively. As such, comparison of diets for impact on *O. insidiosus* developmental time from N1 to adult could only be performed for insects successfully reared on three diet treatments: *Ephestia* eggs, *Ephestia* eggs + *Artemia* cysts at a 1:5 ratio, or *Typha* pollen + *Ephestia* eggs. Among these three diets, total mean nymphal developmental time for *O*. *insidiosus* did not differ significantly (F_2,4_ = 0.178, P = 0.8391) and lasted an average of 12.7 ± 0.3 (mean ± SEM) days for individuals reared on *Ephestia* eggs, 12.3 ± 1.2 days for those reared on *Ephestia* + *Artemia* and 12.3 ± 0.5 days for those reared on a diet of *Ephestia* + *Typha* pollen (Table 2).

**Table 2:** Time required for *Orius insidiosus* predators to complete each developmental instar when reared on various food sources.

Treatment	Developmental time (mean days ± SEM)					
	N1	N2	N3	N4	N5	Total N1-adult
Pollen + *Ephestia*	1.2 ± 0.15	2.6 ± 0.65	3.15 ± 0.70	1.7 ± 0.21	4.2 ± 0.40	12.3 ± 0.49 a
*Artemia* + *Ephestia*	1.1 ± 0.14	2 ± 0.00	3.7 ± 0.00	3.0 ± 0.17	2.7 ± 0.17	12.3 ± 0.17 a
*Ephestia*	1 ± 0.00	1.1 ± 0.09	4.3 ± 0.39	2.8 ± 0.25	3.6 ± 0.36	12.7 ± 1.27 a
*Artemia*	1 ± 0.00	1 ± 0.00	2.3 ± 0.46	5.0 ± 0.0	N/A	N/A
*Typha* pollen	1 ± 0.00	N/A	N/A	N/A	N/A	N/A
No food control	1 ± 0.00	N/A	N/A	N/A	N/A	N/A

Means for total developmental time as shown in the last column were compared using Tukey’s HSD; α = 0.05 (N = 12).

**Figure 2:**
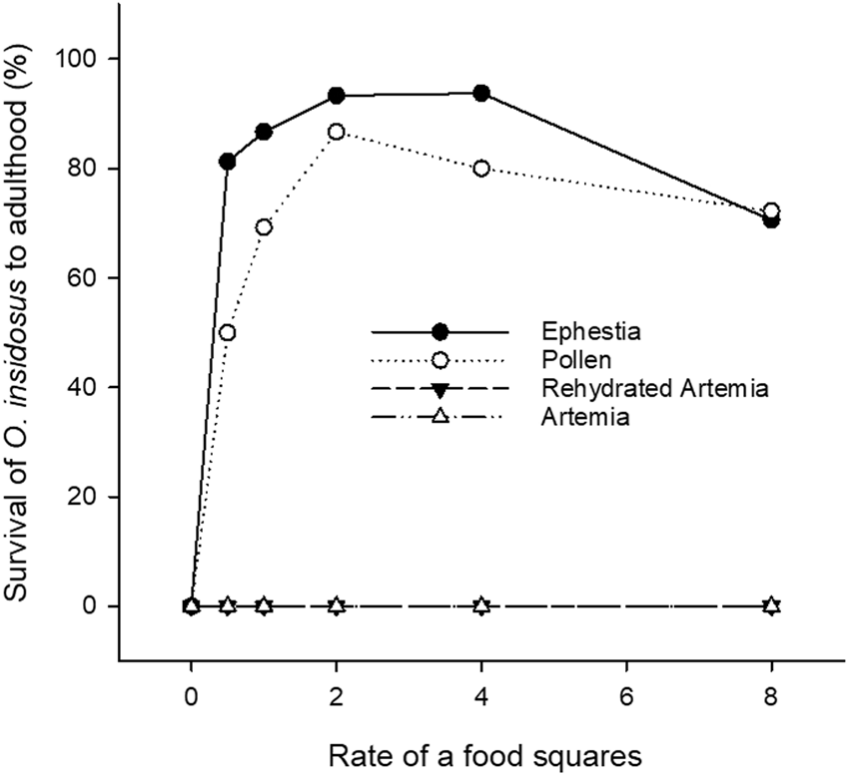
Percent *Orius insidiosus* survival to adulthood. Survival of *Orius insidiosus* through complete nymphal development (N1- adult) when reared on different supplemental diets offered to predators at each of five different rates of 0.5 cm^2^ food squares (N = 12).

In a second round of laboratory trials, mean total nymphal development time was measured for *O. insidiosus* reared on one of four different diet treatments relative to an unfed control with each of the four diets offered to predators at five food square rates (½, 1, 2, 4, and 8 squares). In this trial, no nymphs survived to adulthood on diets of dehydrated *Artemia* cysts, rehydrated *Artemia* cysts and for predators offered these in any of the amounts tested here, as well as for unfed control predators (Fig. 2). As such only *Ephestia* eggs and *Typha* pollen diets, on which predators could develop to adulthood were compared for the impact of food rate on total development time. In a two-way ANOVA, it was found that diet significantly affected developmental time of *O. insidiosus* (F_1,95_ = 75.11, P < 0.0001) but that the amount of food did not. The interaction between diet type and food quantity was not significant. Of the *O. insidiosus* predators offered *Ephestia* eggs, mean nymphal developmental time was of 9.20 ± 1.33 days (N = 67) whereas developmental time on *Typha* pollen was of 10.74 ± 0.18 (N = 53). The proportion of *O. insidiosus* successfully surviving through nymphal development however did peak when offered two food squares, equivalent to 41.84 mg of *Ephestia* eggs and to 7.06 mg of *Typha* pollen on a twice a week basis.

### Greenhouse assays

In greenhouse trials, successful population establishment was achieved for thrips, *Frankliniella occidentalis* (Pergande) and *O. insidiosus* on pepper plants, as well as for greenhouse whitefly, *Trialeurodes vaporariorum* (Westwood),  and *D. hesperus* on tomato plants. No block effect between greenhouse units was observed for any of the analyses performed in this trial (Table 3).

**Table 3:** Analysis of variance for the crop establishment of predators and pests as affected by supplemental diet type, time and greenhouse block as well as their interaction.

*Trialeurodes vaporariorum*	Covariance parameters	Estimate	Z value	Pr > Z
	Greenhouse	0		
	Repetition	0.02648	0.17	0.4309
	Repetition(greenhouse)	0.02732	0.15	0.4408
	diet*repetition(greenhouse)	0.8118	2.16	0.0154
	Residual	5.8772	11.03	<0.0001
	**Fixed effects**	**ndf/ddf** ^a^	**F value**	**Pr > F**
	diet	6/42	13.32	<0.0001
	time	5/245	64.94	<0.0001
	diet*time	30/245	4.19	<0.0001
*Dicyphus hesperus*	Covariance parameters	Estimate	Z value	Pr > Z
	Greenhouse	0		
	Repetition	0.03615	0.97	0.1668
	Repetition(greenhouse)	0		
	diet*repetition(greenhouse)	0		
	Residual	0.6829	11.97	<0.0001
	**Fixed effects**	**ndf/ddf** ^a^	**F value**	**Pr > F**
	diet	6/42	23.56	<0.0001
	time	5/245	66.6	<0.0001
	diet*time	30/245	3.45	<0.0001
*Frankliniella occidentalis*	Covariance parameters	Estimate	Z value	Pr > Z
	Greenhouse	0		
	Repetition	0		
	Repetition(greenhouse)	0.01849	0.29	0.3876
	Diet X repetition(greenhouse)	9.94E-18		
	Residual	2.2429	10.89	<0.0001
	**Fixed effects**	**ndf/ddf** ^a^	**F value**	**Pr > F**
	diet	6/42	77.17	<0.0001
	time	8/392	3.2	0.0015
	diet*time	48/392	7.42	<0.0001
*Orius insidiosus*	Covariance parameters	Estimate	Z value	Pr > Z
	Greenhouse	0.02975	0.64	0.2627
	Repetition	0.01978	0.89	0.1868
	Repetition(greenhouse)	0.001872	0.17	0.4322
	diet*repetition(greenhouse)	0		
	Residual	0.5133	14.18	<0.0001
	**Fixed effects**	**ndf/ddf** ^a^	**F value**	**Pr > F**
	diet	6/42	21.34	<0.0001
	time	8/392	46.69	<0.0001
	diet*time	8/392	2.43	<0.0001

^a^Abbreviations: ndf, numerator degrees of freedom; ddf, denominator degrees of freedom.

#### *Dicyphus hesperus* suppression of greenhouse whitefly

**Figure 3:**
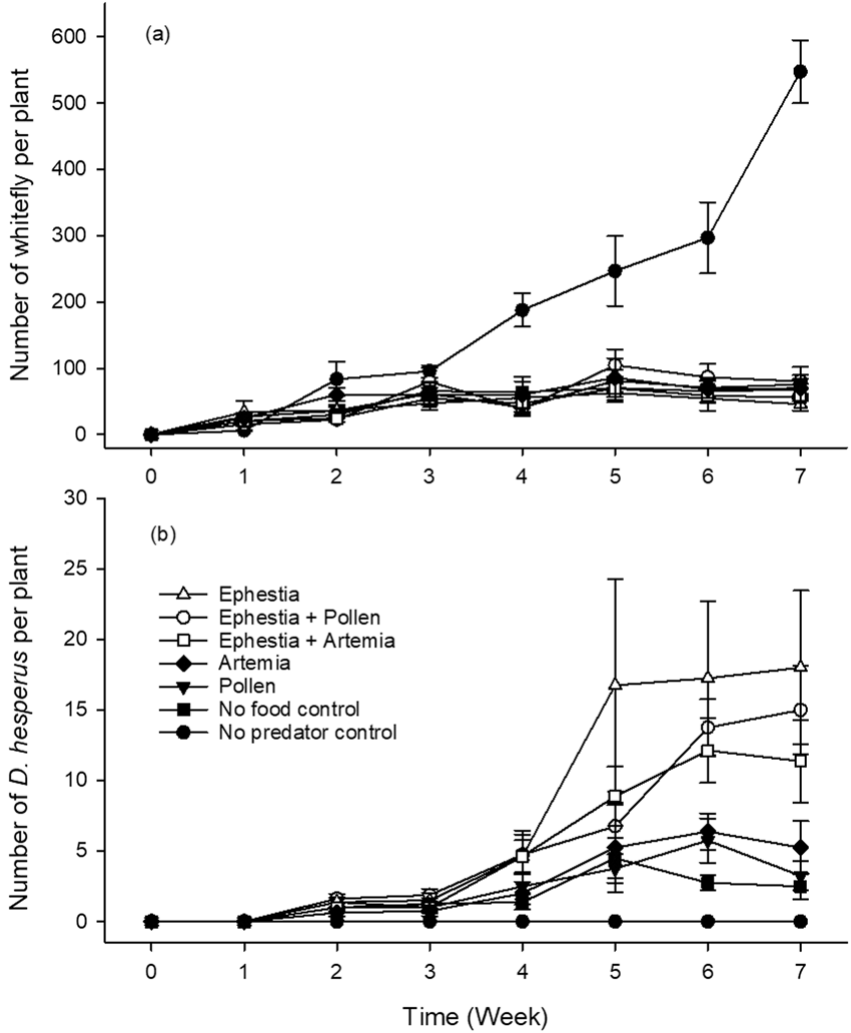
Impact of supplemental foods on whitefly and *Dicyphus hesperus* predator dynamics. Number of greenhouse whitefly, *Trialeurodes vaporariorum* (**a**) and *D. hesperus* predators (**b**) counted per plant weekly in cages designated to one of six supplemental food treatments or a no food control offered to *D. hesperus* predators.

In small cage greenhouse assays, the impact of supplemental foods on *D. hesperus* crop establishment as well as control of the greenhouse whitefly was assessed. In control cages in which *D. hesperus* was not added, a clear expansion of greenhouse whitefly populations was evident by week two of the trial, resulting in a significantly greater amount of whitefly in these cages relative to all other treatment cages (P =< 0.0001; Fig. 3a). An increasing trend of whitefly numbers in the no predator control cages also persisted through to the trial end and starkly contrasted with the low level of whitefly consistently observed in all treatment cages containing *D*. *hesperus* predators. In this trial, all treatments with the presence of *D. hesperus* had weekly whitefly counts that did not exceed an average of 104 individuals per cage, a level achieved on week five in the Ephestia and Pollen treatment.

Over the course of this trial, the number of *D. hesperus* predators per plant increased at markedly different rates depending on the type of food source offered to the predators (Fig. 3b). Notably, predators offered diets including *Ephestia* eggs either alone (18.0 ± 5.45; P = 0.0014) or in combination with pollen (15.0 ± 3.13; P = 0.0044) or *Artemia* cysts (11.4 ± 2.92; P = 0.0311) were significantly more abundant relative to the number in no food control cages. Furthermore, a diet of *Ephestia* eggs alone (P = 0.0023) or in combination with *Artemia* cysts (P = 0.0481) or pollen (P = 0.0072), also led to significantly greater predator numbers relative to the *Artemia* cyst diet alone. In contrast, diets such as *Artemia* cysts alone (5.3 ± 1.86; P = 0.999) and *Typha* pollen alone (3.3 ± 1.05; P = 0.936) produced a similar number of predators relative to the no food control (2.5 ± 0.91; Fig. 3b). It is notable that, among the three top performing diets, the addition of pollen or *Artemia* cysts to *Ephestia* eggs did not improve predator establishment relative to the *Ephestia* eggs diet alone. However, at points such as in week five of the trial, these two supplemental foods alone may have offered some benefit to predators relative to those in the no food control cages (Fig. 3b).

#### *Orius insidiosus* suppression of western flower thrips

**Figure 4:**
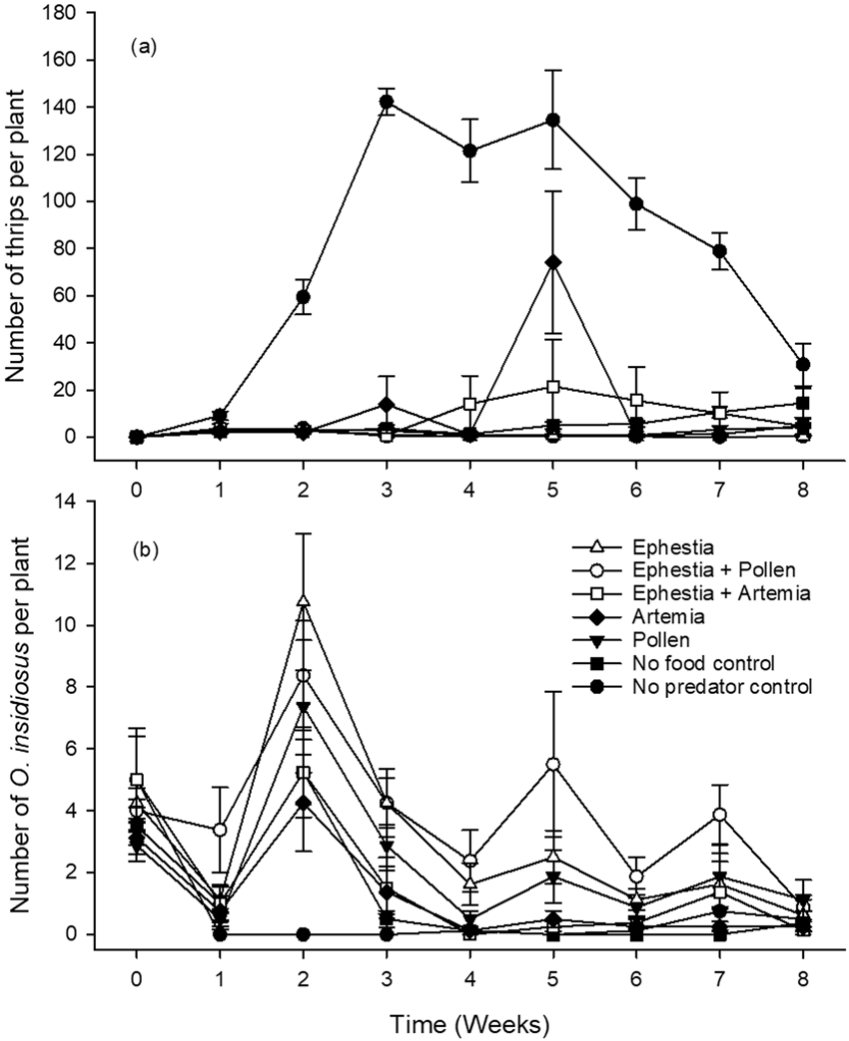
Impact of supplemental foods on thrips and *Orius insidiosus* predator dynamics. Number of western flower thrips, *Frankliniella occidentalis* (**a**) and *Orius insidious* predators (**b**) counted per plant weekly in each cage for each of five supplemental food treatments as well as a no-food control offered to *O. insidiosus* predators.

In greenhouse trials evaluating the impact of diet on *O. insidiosus* population buildup and thrips suppression, a diet by time interaction was observed for analyses of both predator and thrips populations (Table 3). Notably, in all *O. insidiosus* predator containing treatment cages, significant thrips population control was achieved on pepper plants relative to the very high thrips numbers recorded in no predator control cages (P < 0.0001; Fig. 4a). Predator suppression of thrips however, was affected by diet type. This was indicated by the significantly lower total thrips numbers observed in the *Ephestia* egg (P = 0.0222) as well as in the *Ephestia* egg plus *Typha* pollen (P = 0.0162) treatment cages relative to cages in which predators were offered no supplemental food. In addition, though a surge in the number of thrips was apparent in *Artemia* cyst treatment cages in week five of this trial, no overall trial difference in thrips numbers was observed between this treatment and others except for with the no predator control (P < 0.0001; Fig. 4a).

When examining the numbers of *O. insidiosus* predators per plant over time among supplemental food treatments, it was clear that diets that included either *Ephestia* eggs, *Typha* pollen or combinations of these generally supported greater predator numbers relative to other diets (Fig. 4b). For instance, the highest mean number of *O. insidiosus* (3.83 ± 0.62 predators per plant over the course of the trial) was recorded in the *Ephestia* + *Typha* pollen treatment, equivalent to a trial average of 3.2 times more predators in these cages relative to levels in unfed control cages. This treatment was followed in decreasing rank of predator establishment by treatments of *Ephestia* eggs alone (3.09 ± 0.53), *Typha* pollen alone (2.2 ± 0.55), *Ephestia* eggs and *Artemia* cysts (1.65 ± 0.5), *Artemia* alone (1.20 ± 0.43) and finally the no supplemental food control (1.19 ± 0.44). Despite that the addition of pollen to a diet of *Ephestia* resulted in a numerical increase in predators recorded per plant, no difference (P = 0.433) was observed between these two treatments. Clearly, *Typha* pollen alone did not have the same nutritional value as *Ephestia* eggs to *O. insidious,* as the combination of *Ephestia* and pollen resulted in significantly greater predator numbers relative to pollen alone (P = 0.0027). However, the addition of pollen did improve to some degree the survival of predators relative to the unfed control (P = 0.0221). Of all food types tested here, *Artemia* cysts ranked as the least beneficial food source to *O. insidiosus* with no improvement observed in the number of predators per plant relative to the no food control and no improvement either when this diet was added to another type.

## Discussion

The challenge of creating a sustainable augmentative biocontrol program relies on meeting the nutritional requirements for survival and developmental needs of agents applied to crops. This includes mediating the effects of periods of food scarcity and compensating for any nutritional shortcomings of host crops such as the low availability or absence of pollen. Through both highly controlled laboratory assays and more directly translatable greenhouse crop trials, this study served to demonstrate the distinct values of a number of supplemental food types with which it may be possible to achieve greater survival and reproduction of *O*. *insidiosus* and *D*. *hesperus* predators on commercial greenhouse crops. From this study, it is clear that these two predators respond in very different ways to certain food types and these differences will likely influence how food supplementation is applied in commercial settings.

In laboratory and greenhouse assays the developmental time and percent survival of *D*. *hesperus* and *O*. *insidiosus* were investigated under different supplemental food regimens. As expected, it was found that diets that included eggs of *E. kuehniella* invariably sustained the highest survival and shortest nymphal developmental time for both of these predator species. These results are consistent with studies conducted with *M. pygmaeus* for which a diet of *Ephestia* eggs increased the total number of predators on tomato crops relative to control predator population growth^22^ as well as for *O*. *insidiosus* for which this diet resulted in a greater oviposition rate relative to a diet of pollen or no food at all^29^. Eggs of *E*. *kuehniella* are known to have both high protein and high fatty acids contents that together serve to promote predator development and fecundity^24^.

Comparison of alternate food treatments in greenhouse assays with *D. hesperus* showed that predator establishment on any diet that included *Ephestia* eggs, even when it was offered as a 1:5 ratio with *Artemia* cysts offered the greatest level of predator survival. This result, in combination with the relatively comparable nymphal developmental time and survival of predators in laboratory trials involving *Artemia* cysts alone or *Artemia* cysts in combination with *Ephestia* eggs, support the notion that *Artemia* may also be a suitable food that can help support the development and crop establishment of *D. hesperus* when it is combined with a small fraction of *Ephestia* eggs.

This study served to demonstrate the species dependent suitability of much less costly dietary supplements relative to *Ephestia* eggs. As observed in this study, additives such as *Artemia* cysts could in some instances contribute to achieving comparable rates of predator development and survival relative to predators offered an *Ephestia* egg diet. For instance, developmental time of *D*. *hesperus* was significantly shorter on *Artemia* cysts relative to predators reared on pollen or to control unfed predators. Nymphal survival was also good on this food source indicating that it is beneficial to this mirid predator species. In contrast, development of *O. insidiosus* on *Artemia* cysts alone did not result in nymphal survival to adulthood and also tended to prolong nymphal development time for the instars predators that did survive. One factor that could have affected this result was that predators were offered dehydrated as opposed to rehydrated *Artemia* cysts. However in trials performed that examined the value of both dehydrated and rehydrated forms of these cysts to *O. insidiosus* development and survival, it was found that cyst rehydration did not improve either of these parameters, and no predator was able to survive to adulthood on either diet regardless of the amount. Furthermore, the dehydrated form of *Artemia* cysts reflects the state in which the food would typically be applied to a commercial greenhouse crop and so remains the most relevant form to test for its nutritional value to predators. For such dehydrated and decapsulated cysts, the average water content is of approximately 3.39% of the total cyst weight. However it is assumed that these cysts would also absorb ambient water molecules over time following application within a given greenhouse crop. In contrast, other studies done to investigate the value of *Artemia* cysts to *Orius thripoborus* Hesse and *Orius naivashae* Poppius have previously been performed with rehydrated cysts, and in those cases it was found that rehydration could facilitate consumption as it provides a moisture source important to the extraction of nutrients by predators^25^. It is therefore likely that the value of *Artemia* cysts differs considerably for *Orius* predators on a species basis.

As with other mirids, *D*. *hesperus* predators may also have the digestive capacity to allow it to overcome the challenge that a dehydrated *Artemia* cyst may pose. *Dicyphus hesperus* is considered an omnivorous predator, that can undergo trophic switching, able to feed both on plant and animal prey sources and using plant moisture to digest prey animals^14,32^. Mirids, Anthocorids and other phytophagous bugs further use their piercing stylets to secrete digestive enzymes into food sources with the goal of disrupting cell membranes, liquefying contents and extracting the most out of their food^33^. Termed extra-oral digestion, this form of nutrient acquisition may be different enough in efficiency in *O. insidiosus* for digestion of foods so as to render dehydrated *Artemia* cysts less nutritious^25^. Despite this possibility however, the comparable developmental time of *O*. *insidiosus* nymphs when reared on a combined diet of *Ephestia* and *Artemia* at a ratio of 1:5 was statistically similar to individuals reared on *Ephestia* eggs alone, this despite offering predators the same surface area of food in laboratory assays. This indicates that the amount of *Ephestia* eggs being offered to *O. insidiosus* per individual could be scaled back through the use of a combined *Ephestia* egg and *Artemia* cyst formulation, at considerable cost savings to a biocontrol practitioner.

In terms of the economic feasibility of the various supplemental foods, the integration of *Artemia* cysts into systems for rearing and improving predator crop establishment has some merit over the sole use of *Ephestia* eggs. A product currently available in Europe based on cysts of *Artemia* is currently proposed to be about 3% of the cost of pure *Ephestia* (Biobest; Beneficial Insectary, Redding, CA). As a compromise between the two food sources, a formulation including one part *Ephestia* eggs for five parts *Artemia* cysts (Nutrimac^TM^ Biobest, Westerlo, Belgium) is also available in Europe which is nearly one fifth the cost of pure *Ephestia* eggs. With this considerably divergent cost in mind, it is more economically feasible to include this alternative food along with *Ephestia*, particularly as part of a biocontrol program aimed at improving the crop establishment of *D*. *hesperus*.

In laboratory and greenhouse trials, *Typha* pollen alone or in combination with *Ephestia* eggs did not offer any noticeable nutritional benefit for *D. hesperus* predators. As a predominantly leaf feeding and dwelling species, it is not surprising that pollen was not considered a relevant food source for this predator. In contrast however, in laboratory and greenhouse trials, this study showed that *O. insidiosus* survival and development to adulthood could be completed on *Typha* pollen alone, and that a combined diet of *Ephestia* and pollen could have positive implications for the establishment of this predator on crop plants. As a natural food source for multiple anthocorid predators, there is inherent logic to the application of both of these foods together, which may prove to be nutritionally complementary for such a flower dwelling and pollen dependent species as is *O*. *insidiosus*. In a parallel study, corn pollen was found to increase in *O*. *insidiosus* egg production and so possibly other pollen types may also have some value to this predator^34^. However, not all life history parameters of *O. insidiosus* such as fecundity and longevity are improved by including pollen along with lepidopteran food eggs, so the plant origin of the pollen may be an important factor to consider in future studies^35^.

This study demonstrates the importance of assessing the value of supplemental foods at both the laboratory and greenhouse scales, as each contributes to an understanding of attributes unique to each food source and predator species that may not be obvious otherwise. It was shown that *Artemia* cysts could be effectively used to promote *D. hesperus* predator development and that pollen has a role to play in the conservation of *O*. *insidiosus* populations, particularly when applied along with *Ephestia* eggs, or possibly when available as a supplement to pest food resources when available. This knowledge is particularly relevant in the face of growing demand for improved biological control of native crop pest species. It is also relevant in light of a tendency for increased threats by invasive pest species for which generalist predators are frequently seen as a highly adaptable and effective source of biological control^36–39^.

Together, this study plays an important role in demonstrating the function of supplemental foods in improving the resiliency of pest management programs for two important greenhouse crops. They demonstrate the inherent value of such new resources that can be used to counteract pest pressure from unforeseen pest migration events or due to invasive pest introductions. In light of improving the rate of adoption of such supplemental food strategies, future research should be conducted that apply these resources to the commercial greenhouse scale. Trials should also be conducted that focus on identifying the best way to deliver predator foods to the crops without obstructing commercial practices including crop maintenance as well as considerations for pathogen management.

## Methods

### Insect colonies and plants

*Dicyphus hesperus* predators were obtained from GrowLiv (Leamington, ON) as adults aged one week or younger. These predators were reared on whole *Nicotiana tabacum* L. and *Verbascum thapsus* L. plants kept in 0.5 × 0.5 × 1 m screened cages and maintained in a climate controlled chamber set at 24 °C, 55% RH and a 16 L:8D photoperiod (Conviron A1000, Controlled Environments Ltd. Winnipeg, MB). *Dicyphus hesperus* predators were fed eggs of *E. kuehniella* which were applied twice a week directly to plant leaves within the rearing cages. Plants were monitored daily for the emergence of newly hatched (<24 h old) first instar nymphs, which were subsequently, used in laboratory and greenhouse assays.

*Orius insidiosus c*olonies were established from adults supplied by GrowLiv (Leamington, ON). Insects were maintained in a 3 L clear plastic rearing chamber with a mesh-screened lid. Within the rearing chamber, sterile buckwheat hulls served as a niche, and eggs of *E. kuehniella* were provided onto four 2 cm^2^ Post-it™ note strips (3M, London, ON) with the adhesive surface covered in eggs. *Typha* pollen was also offered as a food source that was sprinkled directly into the container. These foods were changed twice a week. The colonies were maintained in a climate controlled chamber set at 24 °C, 55% RH and a 16 L: 8D photoperiod. A water wick made from a glass vial filled with moistened cotton as well as beans of *Phaseolus vulgaris* L. (cv. u1520) served as sources of water, and in the case of the bean, as an oviposition substrate. Fresh beans were provided daily after first being sterilized in a 5% bleach solution for ten minutes. Old beans were removed from the container after a 24 h oviposition period and checked for oviposition marks. Oviposited beans were then maintained in a clear plastic 473 mL cup (Solo®, Merchants Paper, Windsor, ON,) at 24 °C, 55% RH and a 16 L: 8D. Newly hatched (<24 h old) first instar nymphs were subsequently collected and used in laboratory and greenhouse assays.

### Laboratory assays

#### Impact of diet on predator development time and survival

Laboratory trials were conducted to evaluate the effects of various supplemental foods on predatory bug, *D. hesperus* and *O. insidiosus* developmental time and survival. Diet treatments compared in this study included locally-collected fresh-frozen cattail pollen, *Typha latifolia*, flour moth eggs, *E. kuehniella (*Beneficial Insectary Inc., Redding, CA) and decapsulated and dehydrated *Artemia* spp. brine shrimp cysts (Nutrimac^TM^, Biobest, Westerlo, Belgium). In order to test potential complementarity between diet types, additional treatments included combinations of *Artemia* cysts with *Ephestia* eggs at a respective ratio of 5:1 (Nutrimac-Plus^TM^, Biobest, Westerlo, Belgium) as well as a diet of *Ephestia* eggs combined with *Typha* pollen. Each of these five treatments and one unfed control were evaluated for their effects on predator survival and development time through 24 replicated cup assays for trials involving *D. hesperus*, and two sets of 12 replicated Petri dish assays involving *O. insidiosus*, the second of which is described below. Dish assays consisted of a fresh pepper leaf disk embedded in cooled but not yet solidified water agar that was aliquoted into a 5 cm diameter Petri dish. The cup assay consisted of a clear plastic 473 mL cup (Solo®, Merchants Paper, Windsor, ON) which contained a tomato leaflet held in place by a second smaller 59 mL transparent plastic cup (Solo®, Merchants Paper, Windsor, ON), which was pierced at the bottom to accommodate the leaf petiole. Together these two cups formed a space below that accommodated a 40 mL water reservoir for the leaf and a larger chamber above in which predators could be monitored individually through their nymphal development.

Before adding predators to their respective experimental arena, dry treatment diets were bound to the adherent surfaces of 1 cm^2^ or 0.5 cm^2^ plastic Post-it® flags (3M, Maplewood, MN) for trials involving *D*. *hesperus* or *O. insidiosus* respectively^40^. For each 1 cm^2^ flag, the amount of food quantity delivered was measured for a total of ten squares and the average weight (mg ± SEM) is reported as 25.98 ± 1.58 mg for Artemia cysts, 20.92 ± 0.72 mg for *Ephestia* eggs, 13.74 ± 0.65 mg for the mix of *Ephestia* eggs and *Artemia* cysts at a ratio of 1:5 and 3.53 ± 0.16 mg for *Typha* pollen. With the exception of the *Artemia* cyst and *Ephestia* egg combination, combined diets were offered as two separate diet squares at these corresponding weights. Diet squares were replaced at least twice a week to ensure their constant availability and freshness. Insects were transferred individually to experimental arenas, which were then sealed with ventilated plastic lids and maintained in a controlled environment cabinet set at 24 °C, a 16 L: 8D photoperiod and 55% RH. Nymphs of both species were monitored daily for survival, and the timing of moulting events was recorded until the day predators reached adulthood. Statistical analyses were performed to compare the development time of insects maintained on each of the diet treatments. Data were analysed using a one-way ANOVA and means separated using a Tukey’s HSD test at α = 0.05 in SAS (v9.3, Cary, NC)^41^.

#### Impact of *Artemia* cyst rehydration and diet quantity on *Orius insidiosus* development

**Figure 5:**
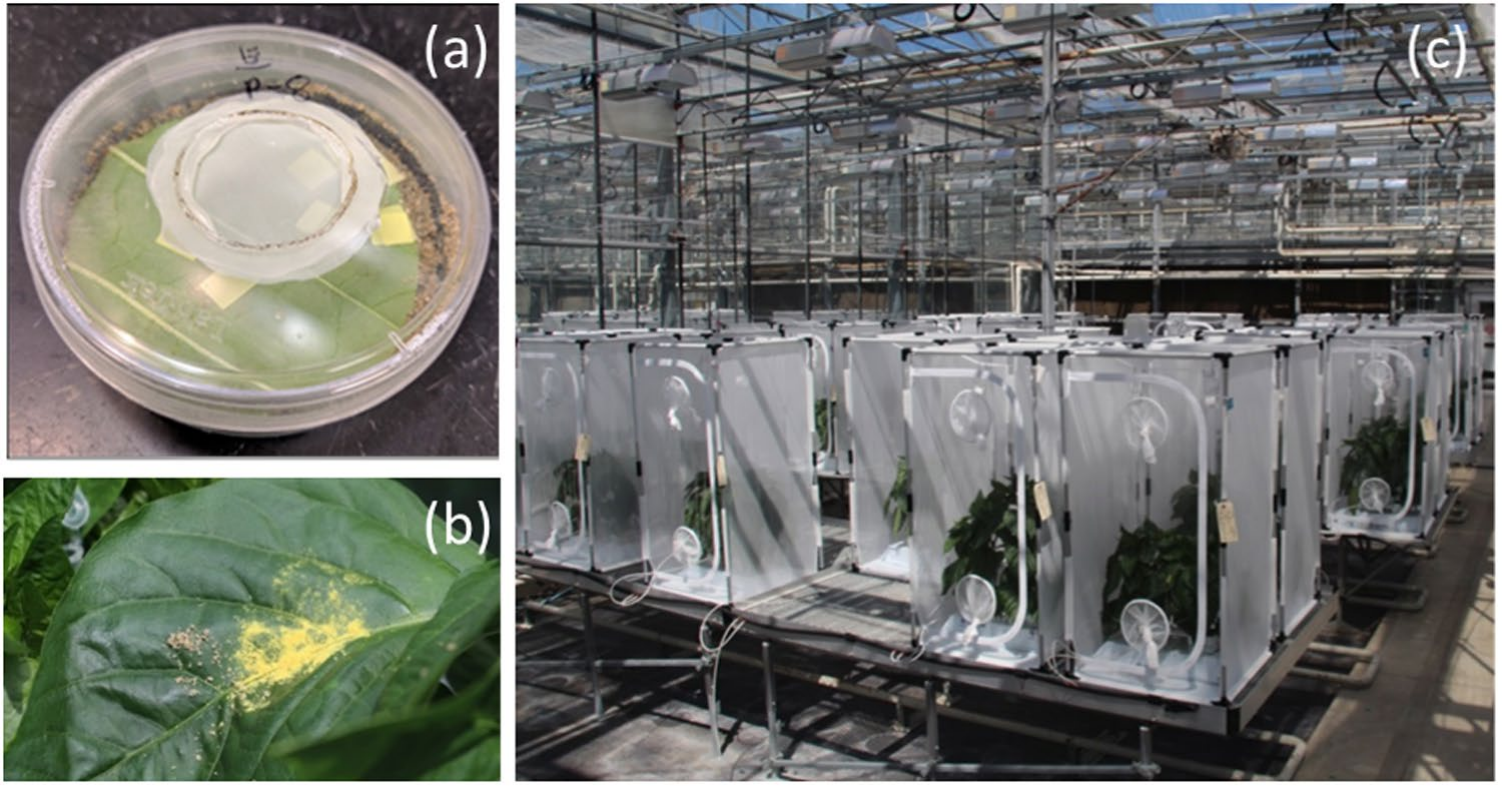
Experimental trial setup. Image of the modified Petri dish experimental arena with a pepper leaf disk embedded within water agar and bare agar surfaces covered in sterile sand to reduce mortality of *Orius insidiosus* nymphs (**a**). Pollen and *Ephestia* egg diet delivered onto the surface of a pepper leaf for greenhouse cage trials involving *Orius insidiosus* (**b**). Greenhouse cage trial setup used to assess *Orius insidiosus* predator survival, rate of population growth and ability of predator to control the western flower thrips, *Frankliniella occidentalis* on pepper plants when offered one of the six supplemental food or control unfed conditions (**c**). Within each of the two greenhouse compartments, cages were randomly assigned to the different treatments.

Initial trials measuring the impact of supplemental diet on developmental time and survival of *O. insidiosus* were complemented by a second set which included modifications as outlined in this section. This was due to the fact that *O. insidiosus* did not survive through nymphal development on a diet of *Typha* pollen alone and dehydrated *Artemia* cysts alone in the first set of trials, despite evidence in the literature for the ability of *O. insidiosus* to successfully complete nymphal development on a diet of *Acer* sp. pollen alone^30^. Also, to investigate the possibility that predator survival was affected by the state of hydration of *Artemia* cysts or the amount of any of the foods offered to *O. insidiosus*, trials were conducted that included these variables as factors. Rehydration was accomplished by soaking the cysts in deionized water for two hours. Once again, diet was provided on a Post-it™ (3M, London, ON) flag cut into 0.5 cm^2^ squares. Rates of ½, 1, 2, 4, and 8 squares per arena were tested for each diet type, as well as a control of no food for each diet. For each of the six rates (0, ½, 1, 2, 4, and 8 squares) and four food type treatments, these trials were replicated a total of 12 times. Diet squares were replaced at least twice weekly. In these trials, areas of bare agar within the Petri dish arena were covered with sterile sand so as to minimize the risk of nymphal mortality due to adhesion to sticky agar surfaces (Fig. 5a). Statistical analyses were conducted for data collected from these trials, consisting of comparison of length of complete *O. insidiosus* nymphal development on the different diet types and amounts through two-way ANOVA and means separation using a Tukey’s HSD test at α = 0.05 in SAS (v9.3 Cary, NC)^41^. For these laboratory analyses data were square root transformed to meet the assumptions of analysis of variance. Homogeneity of variance was also confirmed through graphical visualization of residuals against predicted values.

### Greenhouse trials

In summer and fall 2016, two independent greenhouse trials were conducted, each within two separate greenhouse compartments at the Harrow Research and Development Centre (HRDC) at Agriculture and Agri-Food Canada. Summer trials examined the effects of food supplementation on the establishment of *O. insidiosus* on pepper crops and on the biological control of this predator on western flower thrips, *Frankliniella occidentalis* Pergande (Thysanoptera: Thripidae) (Fig. 5b,c). Fall trials focused on assessing the effects of food supplementation on the establishment of *D. hesperus* on tomato crops and on the biological control of the greenhouse whitefly, *Trialeurodes vaporariorum* Westwood (Hemiptera: Aleyrodidae). In both trials, climate settings in greenhouse compartments consisted of 16 ± 2 °C at night and 24 ± 2 °C in the day with a 16 h photoperiod supplemented as needed with HPS lighting and a RH of 65 ± 5%. Climate data within greenhouse compartment and inside representative cages within each were monitored using HOBO data loggers (Onset Computer Corp., Bourne, MA). Trials were conducted in BugDorm-2400 cages (MegaView Science Co., Ltd. Taichung, Taiwan), which were placed on benches and consisted of experimental units for both trials. All plants in cages were hydroponically irrigated with nutrient media solutions appropriate to crop type.

#### Supplemental food treatment preparation

Prior to the assessment period, supplemental food treatments were assigned to cages according to a randomized complete block design. Cages were labeled with one of six diet treatments as follows: 1. Control no food supplement; 2. *E. kuehniella* eggs (Beneficial Insectary Inc., Redding, CA); 3. *Artemia* cysts (Biobest, Westerlo, Belgium), 4. *Typha* pollen; 5. *Ephestia* eggs, + *Typha* pollen at a rate of one tube of each; 6. *Ephestia* eggs + *Artemia* cysts as a combined 1:5 ratio in a single tube (Biobest, Westerlo, Belgium); 6. No predator and no food supplement control. Supplemental foods were prepared for trials by pre-weighing a 0.1 g quantity of each food type in a 0.5 mL microtube. These were applied to the top of plants using a method appropriate to each food type. For all treatments including either *Artemia* or *Ephestia*, plants were first lightly misted with sterile water using a handheld pump sprayer, then eggs and cysts were distributed on top of moistened leaves so as to prevent food from rolling off plants. For pollen treatments, pollen was directly tapped from the tube, on to the tops of dry leaves (Fig. 5b). Food was replenished onto experimental plants on a weekly basis, directly following plant sampling for predators and pest prey numbers.

#### Impact of diet on *Dicyphus hesperus* greenhouse crop establishment

On August 16, 2016, tomato, *Lycopersicon esculentum* Miller (Solanales: Solanaceae) var. Komeett, was seeded in rockwool plugs and transferred to cubes after two weeks, which were then maintained in transplant greenhouses at the HRDC. Four weeks later, on September 15^th^, these flowering plants in cubes were transplanted onto rockwool half-slabs (Grodan, Roxul Inc., Milton, ON) and placed within trial cages at a rate of two plants per cage. On September 19^th^, six tomato transplants were randomly sampled to determine pre-trial arthropod density. On September 20^th^ (one day post-transplant), greenhouse whitefly, *T. vaporariorum* were introduced onto caged tomato plants at a rate of 20 mating pairs per cage. On September 28^th^ *D. hesperus* were released at a rate of 5 female and 5 male one week old adult predators per cage. On October 5^th^, one week post-predator release, the first non-destructive sampling of whitefly and predators was performed on one of the two plants in each of the trial cages. In order to estimate the number of whitefly within cages, a total of three tomato leaflets, one localized at each of the three canopy layers on the plant (top, middle and bottom), were sampled by gently turning over leaves and counting the number of all life stages. Magnifying visors were used to identify egg and immature stages of the whitefly as well as the number of predated whitefly, which appear as empty whitefly nymphal or pupal cases. For *D. hesperus* predators, a whole leaf was sampled at each of the three plant strata on a weekly basis and predators of all nymphal and adult life stages were counted from both the abaxial and adaxial leaf surfaces. Trials involving *D. hesperus* lasted a total of seven weeks, one week shorter than were pepper trials with *O. insidiosus* due to the faster growth rate of tomato plants in this trial.

#### Impact of diet on *Orius insidiosus* greenhouse crop establishment

On May 2, 2016, seeds of bell pepper, *Capsicum annuum* L. (Solanales: Solanaceae) var. Fascinato, were sown onto rockwool plugs, were transferred to cubes after three weeks and were transplanted onto rockwool half-slabs (Grodan, Roxul Inc., Milton, ON), at a rate of two plants per half-slab. One half-slab was then placed within each of the trial cages, six weeks later on June 13^th^ (Fig. 5). At that time, six of these flowering transplants were randomly sampled to ensure that pre-trial arthropod numbers were low to absent. Briefly, these plants were lightly tapped over a white plastic tray and the number of thrips or other potential insects counted. On June 15^th^, adult female western flower thrips, *F. occidentalis*, were released within each trial cage at a rate of 10 mated females per cage. Thrips were left to establish on plants for a two week period, after which thrips establishment was checked by sampling three cages per greenhouse, once again by lightly tapping plants over a white plastic tray and by counting all thrips from these. Once counted, thrips were placed back onto plants by tapping the tray overtop of the plants. A second release of western flower thrips was repeated on July 26^th^ at a rate of four mated female thrips per cage. On June 22^nd^, 14 *O. insidiosus* predators were released at a rate of seven fifth instar nymphs and seven adults per cage (three female and four male). On this same date, the supplemental food treatments were distributed onto plants as previously outlined. On June 29^th^, one week following the predator release into cages, the first non-destructive sampling for predators as well as for prey thrips was performed. Here also, sampling consisted of lightly tapping plants over a white plastic tray and counting all thrips and predators present. All arthropods were placed back onto plants once counting was completed. Thrips and prey counted included all nymphs and adults of *O. insidiosus* as well as all nymphs and adult thrips. Plants within these trials with *O. insidiosus* and thrips on pepper plants were monitored for a total of eight weeks.

#### Statistical analyses of greenhouse trials

The effects of supplemental diet treatment, time and greenhouse block on the number of predators and target pests per cage in each of the pepper and tomato crop trials were compared through repeated measures ANOVA using the PROC MIXED contrast in SAS (v9.3, Cary, NC)^41^. An autoregressive (AR) covariance matrix was used to define the relationship between time points (Table 3). For these analyses, the total number of western flower thrips, *F.occidentalis* and *O. insidiosus* predators were analyzed from pepper trials and the total number of greenhouse whitefly, *T.vaporariorum* and *D. hesperus* predators were analyzed from tomato trials. Sample units were defined as a cage and the spatial dependence between the cages within each greenhouse was accounted for by including the greenhouse effect as a random block factor in the model. In the case of a significant treatment by time interaction, means separation was performed with Tukey’s HSD. For all analyses involved in greenhouse trials, data were square root transformed to meet the assumptions of analysis of variance. Homogeneity of variance was confirmed through graphical visualization of residuals against predicted values.

## Data Availability

The datasets generated during and/or analysed during the current study are available from the corresponding author on reasonable request.
